# Smoldering Multiple Myeloma: Early Intervention or Structured Surveillance? A Comprehensive Review of Evidence for and Against Early Therapy

**DOI:** 10.3390/cancers18101505

**Published:** 2026-05-07

**Authors:** Kirti Arora, Lara Soueid, Louis Williams, Jahanvi Grover, Diana Basali, Jack Khouri, Yuvraj Kaushal, Sandra Mazzoni, Rockey Dahiya, Beth Faiman, Jason Valent, Faiz Anwer, Shahzad Raza

**Affiliations:** Department of Hematology and Medical Oncology, Taussig Cancer Institute, Cleveland Clinic, Cleveland, OH 44106, USAjahanvigrover10@gmail.com (J.G.); rocksony2859@gmail.com (R.D.);

**Keywords:** smoldering multiple myeloma, early intervention, risk stratification, lenalidomide, daratumumab, measurable residual disease, progression-free survival, surveillance

## Abstract

Smoldering multiple myeloma (SMM) is an asymptomatic plasma cell disorder characterized by a heterogeneous risk of progression to overt multiple myeloma (MM). Historically managed with active surveillance, advances in risk stratification and the development of novel therapeutics have renewed debate regarding early intervention, particularly in patients with high-risk disease. We conducted a narrative review of clinical trials and observational studies evaluating early treatment versus observation in SMM. Outcomes assessed included progression-free survival (PFS), time to progression (TTP), overall survival (OS), treatment-related toxicity, and quality-of-life measures, alongside evolving diagnostic criteria and risk-stratification models that influence therapeutic decision-making. Early randomized trials using conventional cytotoxic therapy did not demonstrate a survival advantage and supported observation as the standard management approach. However, contemporary studies using novel agents have demonstrated improved disease control in selected high-risk populations. The QuiRedex and ECOG-E3A06 trials showed significant reductions in progression risk with lenalidomide-based therapy, and long-term follow-up suggests a potential overall survival benefit in Qui-Redex. The phase III AQUILA trial further demonstrated delayed progression with daratumumab in high-risk SMM using updated diagnostic criteria. Phase II studies evaluating combination regimens, including KRd-based and daratumumab-containing approaches, have reported high response rates and MRD negativity, although survival data remain immature. Importantly, many benefits reflect delayed biochemical or imaging-defined progression rather than prevention of symptomatic end-organ damage. Current evidence supports selective early intervention in carefully defined high-risk SMM populations; however, uncertainties remain regarding long-term survival benefit, optimal treatment duration, quality-of-life impact, and clonal evolution. Active surveillance with modern monitoring remains appropriate for many patients.

## Simple Summary

Smoldering multiple myeloma (SMM) is an asymptomatic plasma cell disorder associated with a variable risk of progression to symptomatic multiple myeloma. Historically, patients have been managed with observation until disease progression. However, advances in risk stratification and the development of novel therapies have renewed interest in earlier treatment for selected high-risk patients. This review examines clinical evidence supporting and opposing early intervention in SMM. While recent trials demonstrate improved disease control with modern agents, uncertainty remains regarding long-term survival benefit and the optimal timing of therapy. Careful patient selection and shared decision-making remain essential when considering early treatment strategies.

## 1. Introduction

### 1.1. Definition

**Figure 1:**
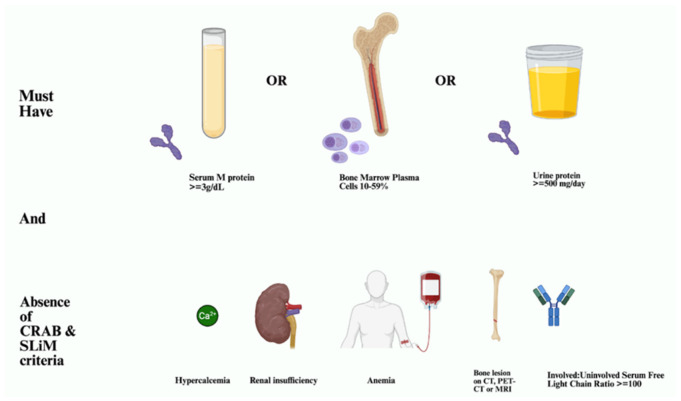
Diagnostic criteria for smoldering multiple myeloma. SMM is defined by serum M-protein ≥ 3 g/dL and/or bone marrow plasma cells ≥ 10% without CRAB features or myeloma-defining events. Adapted from Rajkumar SV et al. International Myeloma Working Group updated criteria for the diagnosis of multiple myeloma [1]. Created with BioRender.com.

Smoldering Multiple Myeloma (SMM) is a precursor plasma cell disorder representing a clinical stage between monoclonal gammopathy of undetermined significance and symptomatic multiple myeloma (MM) [1]. The risk of progression to MM is highest during the first years following diagnosis, estimated at approximately 10% annually during the first five years, decreasing to 3% annually during years 6 to 10 and less than 1% annually thereafter [2]. However, these historical estimates were derived from earlier cohorts evaluated before the incorporation of advanced imaging techniques and updated diagnostic criteria and may therefore overestimate contemporary progression risk. It has now been established that dynamic measures like changing M-protein levels and imaging-based focal lesions are more predictive of progression risk than fixed baseline measures [3]. The diagnostic criteria defining smoldering multiple myeloma are summarized in Figure 1.

### 1.2. Risk Scoring Systems

Given the biological heterogeneity and variable risk of progression, multiple risk stratification tools have been developed to classify patients according to likelihood of disease progression. These models, including the Mayo Clinic model and the International Myeloma Working Group risk stratification system, rely on clinical, laboratory, and cytogenetic parameters such as bone marrow plasma cell percentage, serum free light chain ratio, M protein concentration, and high-risk cytogenetic abnormalities to categorize disease as low, intermediate, or high risk [3]. More recently, genomic and personalized risk prediction approaches have been developed to improve prognostic precision models such as PETHEMA incorporate multiparameter flow cytometry to quantify the proportion of aberrant versus normal bone marrow plasma cells. Ultimately, these criteria are used to categorize the disease as low, intermediate, or high [2,4,5,6]. Accurate stratification is critical for modern management: it guides the intensity of active surveillance and identifies high-risk patients who may benefit from early therapeutic intervention.

The PANGEA model integrates longitudinal clinical and laboratory data using machine learning–based approaches to generate individualized predictions of progression risk in monoclonal gammopathy and smoldering multiple myeloma [4]. Crucially, unlike traditional static risk scores evaluated only at diagnosis, PANGEA continuously updates a patient’s risk profile as new laboratory results become available at successive clinic visits. Furthermore, the model explicitly accounts for the competing risk of non-myeloma-related death, a critical factor in this aging patient population [4] (Table 1).

In addition, whole-genome sequencing studies have identified recurrent genomic alterations and mutational signatures associated with progression from precursor states to symptomatic myeloma [5]. These molecular approaches may refine risk stratification beyond conventional clinical parameters, although prospective validation and integration into routine clinical practice remain ongoing [5].

Despite the availability of several risk stratification models for SMM, they have limited clinical applicability due to variability in predictive performance across populations, variable classification among systems, and a single treatment threshold. These models may not sufficiently describe the change in clinical behavior of the disease, including changing biomarkers or clonal evolution and, risk estimations might not reflect the actual risk of a specific patient, and more personalized and longitudinal methodologies to risk assessment are required.

### 1.3. Management Strategy

Management has historically consisted of active surveillance (Q3–4 months labs and exams, yearly scans) without immediate therapeutic intervention [7]. The central clinical dilemma in SMM lies in distinguishing patients in whom early intervention may delay progression, and impact survival and quality of life. from those in whom treatment represents unnecessary exposure to toxicity without meaningful benefit. However, the evolution of diagnostic criteria, including reclassification of ultra-high-risk SMM as active multiple myeloma by the International Myeloma Working Group (IMWG), has narrowed the contemporary SMM population and altered the interpretation of historical outcomes [2]. Concurrently, modern clinical trials have continually demonstrated significant improvements in progression-free survival and delays in biochemical or imaging-defined progression with early therapy, with a side effect profile similar to that seen in active myeloma [8,9].

Despite these advances, uncertainty persists regarding whether early intervention prevents irreversible end-organ damage, improves overall survival, or meaningfully enhances patient-centered outcomes. Patients are often asymptomatic at diagnosis, which creates a therapeutic dilemma and raises concern about unnecessary exposure to treatment-related toxicity in otherwise clinically stable individuals [10]. As emphasized by Kumar, delaying treatment until disease progression remains a reasonable strategy for many patients with smoldering multiple myeloma, given the absence of definitive survival benefit with early intervention in several studies [10]. The distinction between delaying asymptomatic progression and modifying disease biology remains central to the management debate. This review critically examines the evidence supporting and opposing early treatment in SMM, with particular attention to survival outcomes, surrogate endpoints, treatment-related toxicity, and the evolving role of risk stratification.

## 2. Evidence in Support of Early Treatment in Smoldering Multiple Myeloma

Management of SMM with observation until progression to symptomatic multiple myeloma has traditionally been based on the premise that early treatment may expose patients to unnecessary toxicity without proven survival benefit, as earlier clinical trials did not demonstrate improvement in progression-free survival [11]. This approach reflects recognition that SMM is asymptomatic and that treatment-related harm may occur in the absence of clear clinical gain. However, this conservative strategy has been challenged by emerging clinical and biological data.

Prior to formal recognition of SMM as a distinct entity, Rajkumar et al. evaluated thalidomide in patients with early-stage multiple myeloma and reported an overall response rate of 66%, including a partial response rate of 34% [12]. Significant toxicities were observed, including somnolence, peripheral neuropathy, thrombosis, bradycardia, and edema. Long-term follow-up demonstrated a median overall survival of 86 months and a positive correlation between time to progression and depth of response, with time to progression reaching 61 months among patients achieving partial response [13]. Extended follow-up also revealed cumulative toxicity, including infection, ataxia, and bradycardia [13]. Similar response patterns were reported in additional studies [14,15]. Despite observed responses, high-grade adverse events and the absence of a randomized control group precluded definitive conclusions regarding superiority of early intervention.

A shift toward randomized evaluation occurred with studies assessing therapeutic intervention in biologically high-risk disease (Table 2). Witzig et al. conducted a randomized study comparing thalidomide plus zoledronic acid with zoledronic acid alone in 68 patients [16]. Median time to progression was 2.4 years in the combination group versus 1.2 years in the control group, favoring combination therapy with a hazard ratio of 2.05 and *p* value of 0.02 [16]. Higher rates of adverse events were observed in the treatment group, particularly neuropathy and thromboembolism. Importantly, benefit was limited to delay in progression without demonstration of overall survival improvement. Evaluation of newer agents followed. The interleukin-6 inhibitor siltuximab was investigated in high-risk SMM but did not achieve the anticipated response rate [17].

The QuiRedex trial represented a pivotal randomized study in high-risk SMM, enrolling 119 patients assigned to lenalidomide plus dexamethasone versus observation [9]. At a median follow-up of 40 months, treatment significantly delayed progression to symptomatic multiple myeloma, with median time to progression not reached in the treatment arm compared with 21 months in the observation arm, hazard ratio 0.18 and *p* value less than 0.001 [9]. A 10-year overall survival rate of 94% in the treatment group compared with 80% in the observation group was reported, hazard ratio 0.31 and *p* value 0.03 [9]. The survival benefit reported in the QuiRedex trial, while compelling, must be interpreted with caution in the context of methodological limitations. The observation arm was not mandated to receive lenalidomide-based therapy upon progression to active MM, and consequently, most patients in this group did not receive it. Furthermore, the treatment arm was permitted to intensify therapy at biochemical progression, while the control arm was required to fulfill CRAB criteria before initiating any treatment, creating an asymmetry that may have independently contributed to the observed overall survival advantage. These design features limit direct comparability to modern clinical practice, where highly effective induction regimens are now uniformly available at the time of MM diagnosis.

Long-term follow-up confirmed durable time-to-progression benefit, with 86% of patients in the observation group and 39% in the treatment group progressing to multiple myeloma [18]. Median overall survival had not been reached in either group at that time. Grade 3 adverse events included neutropenia, infection, and rash, each occurring in 6% or fewer patients [18]. At 12.5 years of follow-up, continued benefit in time to progression was observed, 8.5 versus 2.1 years, hazard ratio 0.28 and *p* value less than 0.0001, with sustained overall survival advantage, hazard ratio 0.57 and *p* value 0.032 [19]. However, the trial was conducted exclusively in Spain within PETHEMA centers, which may limit generalizability and introduce potential selection bias. Additionally, variations in post-progression therapy and evolving treatment standards complicate interpretation of survival outcomes.

The ECOG E3A06 trial further evaluated lenalidomide monotherapy versus observation in 182 patients [8]. Lenalidomide significantly improved progression-free survival with hazard ratio 0.28 and *p* value 0.002. Although fewer deaths occurred in the treatment arm, statistical significance for overall survival was not achieved. Grade 3 or higher adverse events included neutropenia and infections and were manageable with dose modification [8]. The phase II study on combination of lenalidomide and elotuzumab reported an overall response rate of 84% and no progression to overt multiple myeloma during follow-up [20]. However, another study demonstrated only modest activity with single agent elotuzumab in SMM [21].

Encouraging results prompted investigation of monoclonal antibodies targeting CD38, including daratumumab and isatuximab [22,23,24]. The phase 2 CENTAURUS trial enrolled 123 patients with intermediate- or high-risk SMM, randomizing them to daratumumab monotherapy across three dosing schedules: extended intense, extended intermediate, or short [22]. Although the co-primary endpoint of a CR rate exceeding 15% was not met across any arm (CR rates of 4.9%, 9.8%, and 0% with longer follow-up for intense, intermediate, and short dosing, respectively), the trial demonstrated meaningful disease control, with 24-month PFS rates of 89.9%, 82.0%, and 75.3% across the three arms [22]. Progressive disease and death rates were lowest in the intense dosing arm, and median PFS exceeded 24 months in all groups [22]. Pharmacokinetic analyses confirmed that the intense schedule-maintained trough concentrations throughout the dosing periods, and no new safety signals emerged [22]. Although the primary efficacy threshold was not achieved, the favorable PFS data and acceptable safety profile provided the rationale for the subsequent phase 3 evaluation of daratumumab in SMM, the AQUILA study [22].

The phase III AQUILA study randomized 390 patients with high-risk SMM to daratumumab versus active monitoring [23]. After a median follow-up of 65.2 months, daratumumab reduced the risk of disease progression or death by 51% compared to observation (HR 0.49; 95% CI, 0.36–0.67; *p* < 0.001), with 5-year progression-free survival rates of 63.1% versus 40.8%, respectively. Notably, the benefit extended to overall survival, with 5-year rates of 93.0% in the daratumumab group versus 86.9% in the active-monitoring group, and a trend toward fewer deaths (7.7% vs. 13.3%; HR 0.52; 95% CI, 0.27–0.98). The treatment was well tolerated; the most common serious adverse event was hypertension, occurring in only 5.7% of daratumumab-treated patients, and treatment discontinuation due to adverse events was low at 5.7%. These results establish daratumumab as the first agent to demonstrate an improvement in overall survival with a favorable trend in high-risk SMM, supporting a paradigm shift away from watchful waiting toward early intervention in this patient population. It remains unclear whether preventing these asymptomatic biochemical milestones improves clinical outcomes or impacts overall survival.

Isatuximab demonstrated partial response or better in more than 64% of patients in a phase II study, with reported quality-of-life improvement [25]. Combination therapy with isatuximab and lenalidomide also produced an overall response rate of 89% and improved quality of life, with myalgia identified as the primary drug-related adverse event [26]. The interim data on phase III ITHACA trial indicate manageable toxicity and very good partial response or better in 73.9% of participants [24]. Ixazomib has also been studied in high-risk SMM, achieving response rates exceeding 50%, although the pre-specified overall response rate of 75% was not met [27].

Multi-drug regimens have also been explored (Table 3). The phase II GEM-CESAR study evaluated induction with carfilzomib, lenalidomide, and dexamethasone followed by autologous stem cell transplantation, consolidation, and maintenance therapy [28]. Designed with a curative intent for high-risk patients, the trial demonstrated highly encouraging efficacy, achieving undetectable measurable residual disease (uMRD) in 62% of patients post-transplant and yielding an overall survival rate of 92% after nearly six years of follow-up, compared to 86.9% in patients with active monitoring in the AQUILA study [23,28]. Biochemical progression occurred in 40% of patients. High-risk cytogenetics predicted progression. Grade 3 and 4 neutropenia occurred in 3% and 5%, respectively, and infection occurred in 10%. Four treatment-related deaths were also reported in the trial, highlighting the potential risks associated with intensive early intervention strategies [28]. A separate phase II study evaluated a similar regimen without transplantation and reported favorable response rates [29].

The ASCENT trial investigated induction with carfilzomib, lenalidomide, daratumumab, and dexamethasone followed by maintenance therapy [30]. 84% of patients achieved MRD at a median of 6.6 months. Three patients experienced progression. Grade 3 or higher hematologic toxicities occurred in 15% and non-hematologic toxicities in 51% [30]. Exclusion of patients with significant comorbidities limits generalizability. Combination therapy incorporating daratumumab, bortezomib, lenalidomide, and dexamethasone has demonstrated an overall response rate of 98% with high MRD negativity in early-phase studies [31]. However, these findings are derived from small cohorts lacking long-term comparative outcomes.

Immunotherapeutic strategies have also been explored (Table 4). A multi-peptide cocktail and PVX-410 vaccine demonstrated anti-myeloma immune responses in SMM [32,33]. A phase 1b study evaluating the addition of the histone deacetylase inhibitor citarinostat assessed immunogenicity in two cohorts: one receiving citarinostat with PVX-410 and the other receiving citarinostat, PVX-410, and lenalidomide [34]. No progression occurred in the doublet cohort, and one patient progressed in the triplet cohort. Increases in CD8+, CD14+, and CD16+ immune cell populations were observed. Most adverse events were grade 1 or 2, including neutropenia, anemia, and injection site reactions [34].

Immune checkpoint inhibition has also been investigated. In a study evaluating pembrolizumab, one patient out of thirteen achieved a stringent complete response, and bone marrow analysis suggested a pre-existing immune profile associated with response [35]. These early immunotherapeutic approaches remain investigational and require larger studies to determine the durability of response and long-term safety. Key clinical trials evaluating early intervention in smoldering multiple myeloma are summarized in Table 1, Table 2, Table 3 and Table 4. Eligibility criteria varied across studies, and definitions of high-risk disease were based on different risk stratification models, including the PETHEMA model, the Mayo Clinic 20/2/20 model, and the International Myeloma Working Group (IMWG) framework.

Collectively, these studies indicate that treatment intervention at an early stage in SMM can slow down the progression and attain deep response, especially in biologically high-risk groups. The size of benefit, however, differs in different studies and seems to be the most significant in trials that are patient-enriched with a higher tumor burden or evolving disease. Significantly, most endpoints are biochemical or imaging-based progression, not clinically significant end-organ harm, and there is debate on whether there is an actual patient-centered advantage to early therapy.

**Table 2:** Randomized trials of early therapy in smoldering multiple myeloma.

Study	Population	Population Criteria (Study Eligibility)	Regimen	Primary Endpoint	Key Result	Major Toxicity
Witzig 2013 [16]	SMM	Asymptomatic (smoldering) multiple myeloma without CRAB features	Thalidomide + ZA vs. ZA	Time to progression	2.4 vs. 1.2 years; *p* = 0.02	Neuropathy, VTE
QuiRedex (2013/2022) [9]	High-risk SMM	PETHEMA	Lenalidomide + dexamethasone vs. observation	TTP; OS	TTP 9.5 vs. 2.1 years; HR 0.57; *p* = 0.032	Neutropenia, infection
ECOG E3A06 (2020) [8]	Intermediate/high-risk SMM	Mayo Clinic	Lenalidomide vs. observation	PFS	HR 0.28; *p* = 0.002	Neutropenia
AQUILA (2025) [23]	High-risk SMM	IMWG	Daratumumab vs. observation	PFS	5-year PFS 63% vs. 41%	Hypertension

**Abbreviations**: SMM, smoldering multiple myeloma; ZA, zoledronic acid; TTP, time to progression; PFS, progression-free survival; OS, overall survival; HR, hazard ratio; VTE, venous thromboembolism.

**Table 3:** Phase II combination and intensive regimens in high-risk SMM.

Study	Population	Population Criteria (Study Eligibility)	Regimen	Response Outcome	Notable Findings	Major Toxicity
GEM-CESAR (2024) [28]	High-risk SMM	PETHEMA or Mayo risk models	KRd + ASCT + maintenance	uMRD 62% post-transplant	40% biochemical progression	Infection (10%), neutropenia (grade 3–4: 8%)
ASCENT (2024) [30]	High-risk SMM	Mayo or IMWG	Dara-KRd	ORR 97%; MRD negativity 84%	3 progressions reported	Hematologic (15%); non-hematologic (51%)
Kazandjian (2023) [29]	High-risk SMM	Mayo or PETHEMA	KRd + maintenance	74% MRD-negative CR	9.3% progression	Thromboembolism, cardiac events
Nadeem (2024) [31]	High-risk SMM	IMWG 2020, Mayo 20-2-20, PETHEMA or high-risk cytogenetic features	D-RVD	ORR 98%; 65% MRD-negative	1 biochemical progression	Neutropenia
Mailankody (2022) [27]	High-risk SMM	Mayo or PETHEMA	Ixazomib + dexamethasone	ORR 57%	21% progression	AKI, fatigue

**Abbreviations**: SMM, smoldering multiple myeloma; KRd, carfilzomib/lenalidomide/dexamethasone; ASCT, autologous stem cell transplantation; Dara, daratumumab; D-RVD, daratumumab/bortezomib/lenalidomide/dexamethasone; ORR, overall response rate; MRD, measurable residual disease; uMRD, undetectable MRD; CR, complete response; AKI, acute kidney injury.

**Table 4:** Monoclonal antibody and immunotherapy approaches in SMM.

Study	Population	Population Criteria (Study Eligibility)	Therapy	Key Outcome	Clinical Impact	Major Toxicity
CENTAURUS (2020) [22]	Intermediate/high-risk SMM	IMWG	Daratumumab	24-month PFS up to 84%	Led to phase III AQUILA	Fatigue, URTI
ISAMAR (2023) [26]	High-risk SMM	PETHEMA or Mayo	Isatuximab ± lenalidomide	ORR 89%	Improved patient-reported anxiety	Myalgia, neutropenia
Manasanch (2019) [25]	High-risk SMM	PETHEMA	Isatuximab	≥PR 62.5%	No deaths reported	Headache, mucositis
PVX-410 (2025) [34]	SMM	SMM with measurable disease; not restricted to high-risk populations	Vaccine ± lenalidomide	Immune activation; minimal progression	Proof-of-concept immunotherapy	Injection site reactions
Pembrolizumab (2019) [35]	SMM	Intermediate/high-risk SMM with measurable disease and no myeloma-defining events	Checkpoint inhibitor	1 sCR	Immune profiling insights	Limited data

Abbreviations: SMM, smoldering multiple myeloma; PFS, progression-free survival; ORR, overall response rate; PR, partial response; sCR, stringent complete response; URTI, upper respiratory tract infection.

## 3. Evidence Against Early Treatment in Smoldering Multiple Myeloma

Randomized clinical trials conducted prior to the era of novel agents did not demonstrate an overall survival advantage with early therapeutic intervention in smoldering multiple myeloma and established active surveillance as the standard management approach for asymptomatic patients.

Melphalan-prednisone therapy was evaluated in early randomized studies of asymptomatic stage I disease. Studies by Hjorth et al. and Riccardi et al. observed no difference in overall survival [11,36]. Although these historical studies formed a basis of observation, it is not clear how the studies will be applicable in the age of new agents.

Biologic heterogeneity further complicates interpretation of early intervention. Rosinol et al. characterized evolving and non-evolving subtypes of SMM based on M-protein kinetics [37]. The evolving subtype was associated with shorter time to progression, 1.3 versus 3.9 years, *p* value 0.007, yet overall survival did not differ significantly between subgroups [37]. Among patients who required chemotherapy, a substantial proportion demonstrated limited response [37]. These observations underscored variability in disease biology and the difficulty of identifying individuals who would derive durable benefit from preemptive therapy.

Pharmacologic strategies targeting skeletal disease were also investigated. In the GIMEMA trial, zoledronic acid did not reduce progression risk or prolong time to progression compared with observation, despite a reduction in skeletal-related events [36]. A long-term study with pamidronate confirmed absence of effect on overall survival [38]. These data indicate that bisphosphonates reduce skeletal morbidity but do not modify the underlying disease course in SMM.

Cytogenetic and genomic profiling studies have refined risk stratification but have not established therapeutic advantage for early intervention. High-risk abnormalities, including del(17p13), t(4;14), and +1q21, are associated with shorter time to progression and inferior survival outcomes [39,40]. However, these findings derive from observational cohorts without interventional comparison. Prognostic enrichment does not equate to demonstrated benefit from early treatment. Emerging genomic approaches may further refine risk stratification in smoldering multiple myeloma. Next-generation sequencing and whole-genome analyses have identified recurrent molecular alterations and patterns of clonal evolution associated with progression to symptomatic disease [5]. These genomic insights may allow more precise identification of patients at the highest risk of progression, although their integration into routine clinical decision-making remains under investigation. In parallel, advances in immunotherapy have introduced cellular therapies such as chimeric antigen receptor (CAR) T-cell therapy, which have demonstrated deep and durable responses in advanced disease. While these approaches raise the possibility of curative strategies in plasma cell disorders, their role in asymptomatic precursor conditions such as SMM remains investigational.

A further argument against universal early intervention relates to the biological heterogeneity captured incompletely by existing risk models. The three major stratification systems: PETHEMA, Mayo Clinic 20/2/20, and the IMWG model, which rely on different combinations of clinical, laboratory, and cytogenetic parameters and do not consistently classify the same patients as high-risk [3,6]. As a result, the populations enrolled in the QuiRedex trial (selected by PETHEMA criteria requiring ≥95% aberrant plasma cells and immunoparesis), the ECOG E3A06 trial (intermediate and high-risk by Mayo Clinic factors), and the AQUILA trial (high-risk by Mayo 2018 or IMWG criteria incorporating cytogenetics) are not directly comparable [9,10,27]. Observed treatment benefits may therefore reflect the enrichment of different biological subgroups rather than a generalizable effect across the broader SMM population, and cross-trial comparisons of efficacy must be interpreted with this limitation in mind.

Collectively, historical evidence indicates that early intervention in asymptomatic SMM did not improve overall survival when evaluated with conventional cytotoxic regimens. Although contemporary therapies possess improved tolerability profiles, the absence of consistent survival benefit in earlier trials established the principle that treatment initiation in asymptomatic individuals requires evidence of meaningful clinical advantage beyond delay of biochemical progression.

## 4. Discussion

The management of smoldering multiple myeloma (SMM) has evolved substantially over the past two decades, driven by refinements in diagnostic criteria, risk stratification models, and therapeutic options [1,2,3]. Historically defined by serum M-protein ≥ 3 g/dL and bone marrow plasma cells ≥ 10% in the absence of CRAB features, SMM encompassed a biologically heterogeneous population in whom early intervention failed to demonstrate survival benefit when cytotoxic therapies were used [11,41]. A pivotal shift occurred in 2014 with the introduction of myeloma-defining biomarkers by the International Myeloma Working Group [1]. Patients with ≥60% clonal plasma cells, free light chain ratio ≥ 100, or focal MRI lesions were reclassified as symptomatic multiple myeloma requiring therapy [1]. Subsequent analyses have reevaluated the predictive value of these biomarkers, suggesting that progression risk among patients meeting these criteria may be more heterogeneous than initially reported [42]. This reclassification has also introduced lead-time bias into subsequent trial interpretation: patients enrolled as “high-risk SMM” in pre-2014 studies would, in part, now meet criteria for active myeloma and require immediate treatment. Apparent benefits of early intervention in those historical cohorts may therefore partly reflect earlier initiation of therapy in patients already meeting criteria for active disease, rather than a genuine modification of smoldering disease biology.

Modern randomized trials such as ECOG E3A06 and QuiRedex demonstrated significant delays in progression with lenalidomide-based therapy [8,9]. More recently, the AQUILA study extended these findings using daratumumab [23]. This also provided the basis for the November 2025 FDA approval of subcutaneous daratumumab for high-risk SMM, which is the first regulatory approval for a therapeutic indication in this disease stage [23]. Clinicians must now navigate the gap between regulatory approval based on a progression-free survival endpoint and the absence of a statistically significant overall survival benefit [23], particularly when counseling asymptomatic patients about initiating indefinite therapy. As treatments for active myeloma continue to improve, demonstrating that early treatment of SMM prolongs survival becomes increasingly difficult, since patients who progress and receive modern therapy at that point may also have favorable outcomes. Whether delaying biochemical progression translates into improved long-term survival or preserved quality of life remains uncertain. Furthermore, given the rapid pace of therapeutic advances in myeloma, agents under investigation in long-term SMM trials today risk being superseded by more effective treatments before trial results become available, potentially rendering their findings clinically obsolete.

Measurable residual disease negativity has emerged as an attractive surrogate endpoint in phase II trials employing multi-agent regimens [25,32,34]. Unlike in active multiple myeloma, where MRD negativity has been prospectively validated as a surrogate for progression-free and overall survival across multiple large trials, no equivalent validation exists in the SMM setting. Achieving MRD negativity in a patient who may never have developed symptomatic disease cannot be equated with therapeutic cure, and the use of MRD negativity to justify prolonged intensive therapy, including autologous transplantation, in an asymptomatic individual warrants considerable caution until prospective outcome data mature. Treatment burden must also be weighed carefully. Even relatively well-tolerated regimens such as lenalidomide or daratumumab require prolonged administration, exposing patients to cumulative risks including hematologic toxicity (such as neutropenia, anemia, and thrombocytopenia), infection, thromboembolism, and hypertension [8,23]. Intensive regimens incorporating proteasome inhibitors, monoclonal antibodies, and autologous transplantation demonstrate impressive response rates but remain investigational, with limited long-term survival data [25,32,34]. An additional concern with early intensive therapy is the theoretical risk of inducing clonal selection. Repeated cycles of proteasome inhibitors and immunomodulatory agents under curative-intent protocols may exert selective pressure on the plasma cell clone, potentially enriching cytogenetically high-risk subclones at relapse and narrowing subsequent therapeutic options. While this has not been formally demonstrated in the SMM context, it echoes patterns observed in relapsed and refractory active myeloma and merits prospective evaluation. Alternative strategies such as enrolling high-risk SMM patients in trials of non-daratumumab-based regimens, or reserving daratumumab for those at the highest end of the risk spectrum deserve consideration as the field moves forward.

Patient-centered outcomes deserve greater emphasis. Although some studies report reduced anxiety with active treatment [26], SMM remains asymptomatic at diagnosis, and chronic therapy introduces its own psychological and physical burdens. Observational data from population-based cohorts further suggest increased infection risk in SMM, underscoring underlying immune dysregulation [43]. Risk stratification models have improved prognostic precision [2,3], yet inter-model variability and lack of universal validation limit their ability to define a uniform treatment threshold. Personalized progression modeling such as PANGEA represents a promising approach but requires prospective validation [4].

Taken together, current evidence supports considering early daratumumab monotherapy in high-risk SMM defined by contemporary criteria, particularly in younger, fit patients where downstream access to daratumumab-based induction at progression to MM can be ensured, in consideration with patient preference and insurance-based access. Prospective data on outcomes following prior daratumumab exposure and trials incorporating optimized salvage therapy in the control arm are needed before this approach can be broadly recommended [8,23]. The central unanswered question remains whether early therapy modifies disease biology and ultimate survival or merely advances treatment exposure without altering the natural history. This may be addressed by a more rational approach to treatment selection in SMM, by longitudinal monitoring of disease trajectory rather than static baseline risk classification alone. The PANGEA model, which generates continuously updated progression risk estimates from serial laboratory data while accounting for competing non-myeloma mortality risk, offers a promising framework to identify patients whose rising trajectory warrants early intervention while sparing those with stable disease [4].

Future patient selection for early intervention in SMM will likely require integration of molecular, immune, and clinical data beyond current risk models. Whole-genome sequencing has demonstrated that progressive SMM harbors a distinct mutational landscape, including driver mutations in RAS pathway genes, 1q gain, and del(17p), that may be detectable before clinical progression, enabling genomically informed risk stratification [5,40]. Monitoring circulating tumor DNA offer the prospect of tracking clonal evolution non-invasively between clinic visits. Among emerging clinical biomarkers, serum B Cell Maturation Antigen (BCMA) is particularly promising, with elevated and rising sBCMA levels being associated with imminent progression to active MM [44,45]. Given the accessibility of these markers as a blood-based assay, these could readily be incorporated into dynamic monitoring frameworks such as PANGEA. Ultimately, integration of these multi-omics layers with longitudinal clinical data within machine learning-based platforms represents the most promising direction for identifying the subset of SMM patients who genuinely require early intervention, while sparing those with biologically stable disease from unnecessary treatment.

## 5. Conclusions

The threshold for initiating therapy in smoldering multiple myeloma must remain grounded in demonstrable clinical benefit. While contemporary randomized trials in selected high-risk populations show meaningful reductions in progression, consistent and generalizable evidence of long-term survival advantage or prevention of irreversible organ damage remains limited.

Based on current evidence, early intervention with daratumumab monotherapy should be considered in high-risk SMM patients defined by the IMWG criteria who demonstrate a rising disease trajectory on serial monitoring, balancing the benefit of preventing organ damage against the risk of compromising future daratumumab-based therapy at MM progression. Future efforts should focus on integrating genomic data, immune profiling, and emerging biomarkers such as serum BCMA within dynamic risk prediction platforms to better identify patients who truly require early treatment.

Ongoing studies are also evaluating whether earlier application of advanced immunotherapies, including chimeric antigen receptor (CAR) T-cell therapy and bispecific antibodies, may modify disease biology in high-risk smoldering multiple myeloma. For example, the ECOG-ACRIN DETER-SMM trial is currently investigating early intervention strategies incorporating daratumumab in patients with high-risk SMM [46]. This again warrants caution with use due to concerns of treatment-related toxicity and treatment resistance if case of progression of the disease in the future.

Future investigations should prioritize endpoints that directly reflect patient benefit, including overall survival, quality of life, functional preservation, and post-progression outcomes. Clarification of optimal treatment duration, feasibility of MRD-guided discontinuation, and the impact of early intervention on clonal evolution will be essential.

Until such data mature, early therapy in SMM should be individualized, informed by validated risk assessment, preferentially in patients with high-risk SMM by validated criteria and aligned with patient preference.

## Figures and Tables

**Table 1:** Risk stratification models of SMM.

Model	Key Parameters	Risk Categories	Comments
Mayo clinic [2]	BMPC ≥ 10%, serum M protein ≥ 3 g/dL, serum FLC ratio ≥ 8 (but <100); 20/2/20 rule	Low, Intermediate and High	Widely adopted
IMWG [3]	BMPC ≥ 10%, serum M protein ≥ 3 g/dL, serum FLC ratio ≥ 8 (but <100); 20/2/20 rule, cytogenetics (del17p, t(4;14), t(14;16), 1q gain)	Low, Intermediate and High	Widely adopted, Incorporates cytogenetics
PETHEMA [6]	Proportion of aberrant plasma cells among total BMPC by MFC; immunoparesis	Low, Intermediate and High	Requires specialized MFC laboratory expertise
PANGEA [4]	Serum M protein concentration, FLC ratio, age, creatinine concentration, BMPC% and hemoglobin, with FISH, and BMPC% & FISH can also be included depending on model	Individualized Low, Intermediate and High continuous score	Personalized and dynamic

## Data Availability

No new data were created or analyzed in this study.
